# Class III Correction With Reverse Twin Block—A Case Report

**DOI:** 10.1002/ccr3.9700

**Published:** 2024-12-19

**Authors:** Sushant Pandey, Rajesh Gyawali, Prabhat Ranjan Pokharel, Avinash Chaudhary, Samikshya Sangroula

**Affiliations:** ^1^ Department of Orthodontics and Dentofacial Orthopaedics BP Koirala Institute of Health Sciences Dharan Nepal

**Keywords:** class III malocclusion, early correction, removable appliance, reverse twin block

## Abstract

The orthodontic management of patients with Class III malocclusion poses numerous treatment challenges. Various removable, fixed, orthopedic, and myofunctional appliances have been recommended for its correction. The Reverse Twin Block is a removable appliance which has been used for the early management of such cases. A 10‐year‐old female with no significant medical and family history presented with the chief complaint of forward placement of lower front teeth on biting. Extraoral examination revealed a mesoprosopic face, a prominent lower lip, and a shallow mentolabial sulcus. Facial profile was straight at rest and concave at maximum intercuspation. Intraoral examination revealed a Class III incisor relationship with premature contact between 21 and 31, causing a forward path of closure of the mandible. The patient had a reverse overjet of 2 mm, an overbite of 5 mm, and a bilateral Super‐class I molar relationship at maximum intercuspation. Lateral cephalogram analysis revealed a mild skeletal class III denture base (ANB = 0°, Wits = −4 mm) due to a prognathic mandible (SNB = 82°). The treatment objectives included elimination of the premature contact, establishment of a positive overjet, and improvement of the facial profile. A Reverse Twin Block appliance was planned and prescribed for full‐time wear for 8 months. At the end of treatment, the patient had a Class I incisor relationship, straight facial profile, Class I molar relationship on the left side, and Super‐class I molar relationship on the right side. The Reverse Twin Block is a simple and well‐tolerated appliance that can effectively correct developing Class III malocclusion in carefully selected cases.

## Graphical abstract

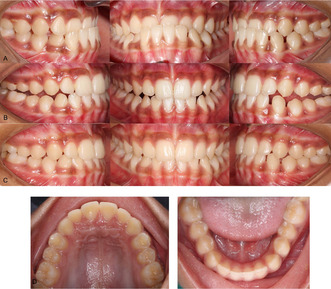

**Summary**

- The Reverse Twin Block appliance is effective in treating mild Class III malocclusion.
- The effects are primarily dentoalveolar: proclination of the maxillary incisors and retroclination of the mandibular incisors.
- It is well‐tolerated and cost‐effective. However, patient compliance and careful case selection are crucial for successful outcomes.

## 1 Introduction

Class III malocclusion presents significant management and prognostic challenges due to the complex interplay of environmental and innate factors in its etiology [1]. It often manifests as mandibular prognathism, maxillary retrognathism, or a combination of both. The multifactorial nature of this condition encompasses genetic heredity, environmental influences, and their combinations, potentially involving factors such as tongue size or position, atypical swallowing patterns, early loss of deciduous teeth, trauma, tonsil hypertrophy, and oral habits [2, 3, 4].

The timely management of Class III malocclusion is essential in order to redirect jaw growth favorably, normalize occlusion, as well as to simplify and reduce future comprehensive orthodontic or surgical treatment needs [5]. Various treatment modalities have been described in the literature, including removable myofunctional appliances, fixed appliances, chin cups, protraction headgears, and skeletal anchorage systems [2]. The Reverse Twin Block (RTB) appliance, introduced by Clark [6], has emerged as a simple, comfortable, and efficient functional appliance for correcting developing Class III malocclusions. The RTB is a removable functional appliance that effectively corrects Class III incisor relationships and anterior displacements through sagittal correction, primarily achieved by proclining the maxillary incisors and retroclining the mandibular incisors [6]. While its efficacy has been demonstrated in mixed dentition [7, 8], limited literature exists on its application in permanent dentition for skeletal Class III malocclusions [9].

This case report describes the successful treatment of a skeletal Class III malocclusion in permanent dentition using a RTB appliance. By presenting this case, we aim to contribute to the growing body of evidence supporting the versatility and effectiveness of this appliance in managing Class III malocclusions beyond the mixed dentition stage.

## 2 Case Presentation

**FIGURE 1:**
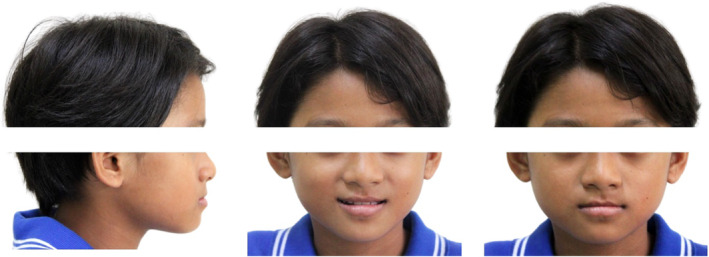
Pre‐treatment extraoral photographs.

A 10‐year‐old female with no significant medical history presented with the chief complaint of forward placement of lower front teeth on biting. There was no history of a similar problem in her family. Extraoral examination revealed a mesomorphic build, mesoprosopic facial form, a prominent lower lip, a shallow mentolabial sulcus, and normal nasolabial angle. Her face was apparently symmetrical (Figure 1). Facial profile was straight at rest and concave at maximum intercuspation. Examination of the temporomandibular joint revealed no abnormality. Deglutition, speech, and mastication were normal. No oral habits were present.

**FIGURE 2:**
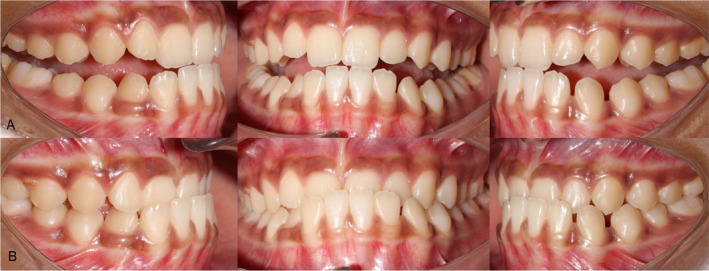
Intraoral photographs at the most retruded position (A) and at maximum intercuspation showing forward displacement of the mandible (B).

**FIGURE 3:**
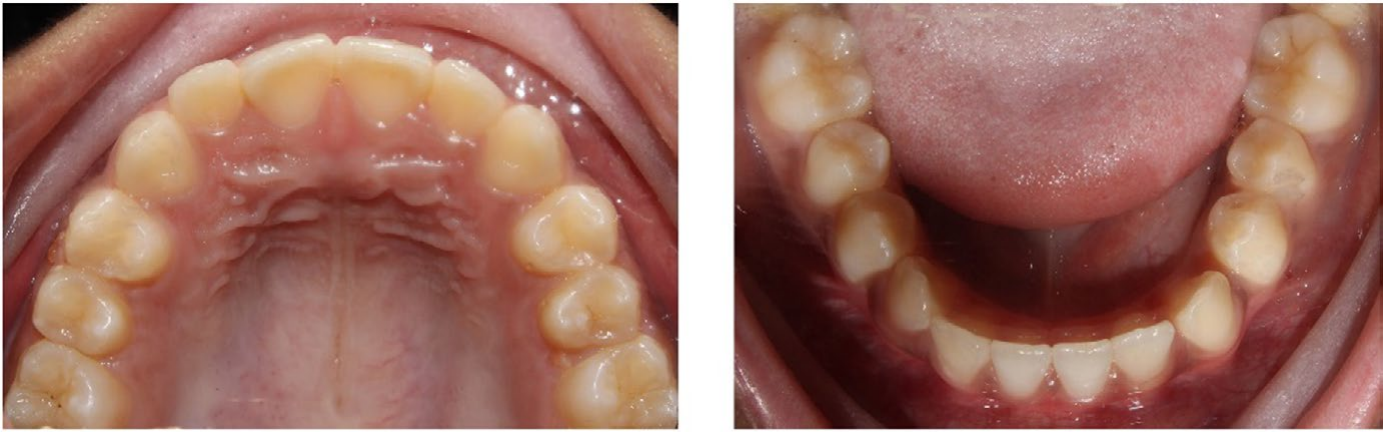
The maxillary and the mandibular arches (Pre‐treatment).

**FIGURE 4:**
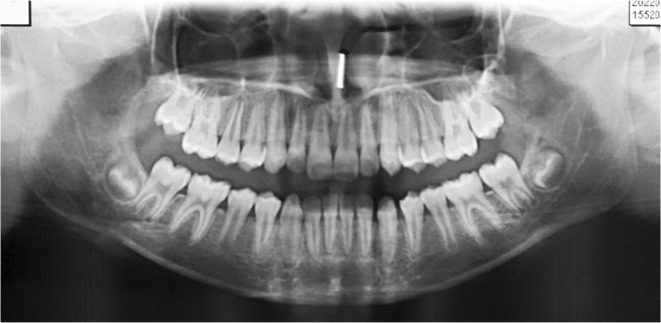
The orthopantomogram of the patient.

Intraoral examination revealed a healthy gingival and periodontal status with no carious teeth. The patient had a forward path of closure of the mandible due to premature contact between 21 and 31, causing anterior cross‐bite. The patient had a reverse overjet of 2 mm, an overbite of 5 mm, and a bilateral Super‐class I molar and a Class III incisor relationship at maximum intercuspation. The maxillary arch was well aligned with no crowding or spacing (Figures 2 and 3). The mandibular arch was also well aligned with a space of 1 mm present both mesial and distal to the mandibular left canine. The depth of the curve of Spee was 2.5 mm. Orthopantomogram revealed developing tooth buds of mandibular third molars and missing tooth buds of maxillary third molars (Figure 4).

**FIGURE 5:**
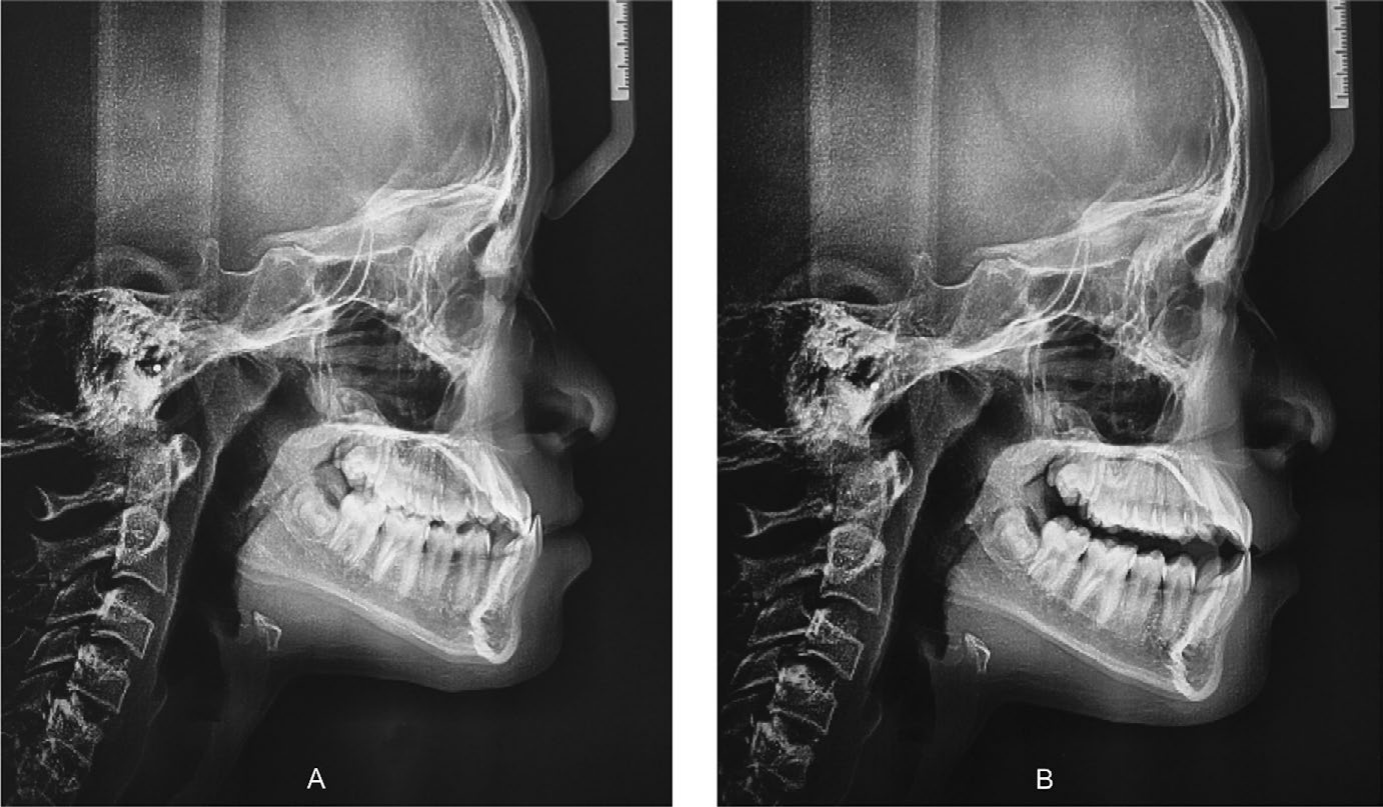
Lateral cephalograms taken at the intercuspal position (A) and at the retruded contact position (B).

Lateral cephalograms were taken at intercuspal position (ICP) and retruded contact position (RCP) (Figure 5). The correction suggested by Lam et al. [10] was applied to the parameters affected by bite opening in the cephalogram taken at RCP. Bite opening was 5 mm.

The skeletal parameters suggested that the patient had a mild skeletal class III denture base (ANB = 0°, Wits = −4 mm) due to a prognathic mandible (SNB = 82°). The growth pattern of the patient was hypodivergent (FMA = 23°, SN‐GoGn = 27°). The maxillary incisors were proclined (U1‐NA = 31°), whereas the lower incisors were slightly retroclined (L1‐NB = 22°). The nasolabial angle was normal (Table 1). Forward displacement of the mandible due to premature occlusal contact causing anterior cross‐bite is a characteristic of both pseudo‐Class III and true skeletal Class III malocclusion [11]. However, in pseudo‐Class III malocclusion, the patient has normal maxilla and mandible with skeletal Class I denture base [12].

**TABLE 1:** Pre‐treatment cephalometric parameters at ICP and RCP, and post‐treatment cephalometric parameters.

Parameters	Normal range	Intercuspal position	Retruded contact position^a^	Post‐treatment
SNA	82°	82°	—	82°
SNB	80°	84°	82°	82°
ANB	2°	−2°	0°	0°
Wits appraisal	0 mm	−6 mm	−4 mm	−4 mm
FMA	25°	23°	—	26°
SN‐GoGn	32°	27°	—	30°
U1‐NA	22°/4 mm	31°/6 mm	—	34°/6.5 mm
L1‐NB	25°/4 mm	23°/6 mm	22°/5 mm	19°/3 mm
IMPA	90°	89°	—	85°
Nasolabial angle	102° ± 8°	107°	—	105°

Abbreviations: ANB, Point A‐Nasion‐Point B; FMA: Frankfort Mandibular Plane Angle; IMPA: Incisor Mandibular Plane Angle; SNA, Sella‐Nasion‐Point A; SNB, Sella‐Nasion‐Point B.

^a^ For the cephalogram at RCP, only the parameters affected by bite opening are mentioned.

**FIGURE 6:**
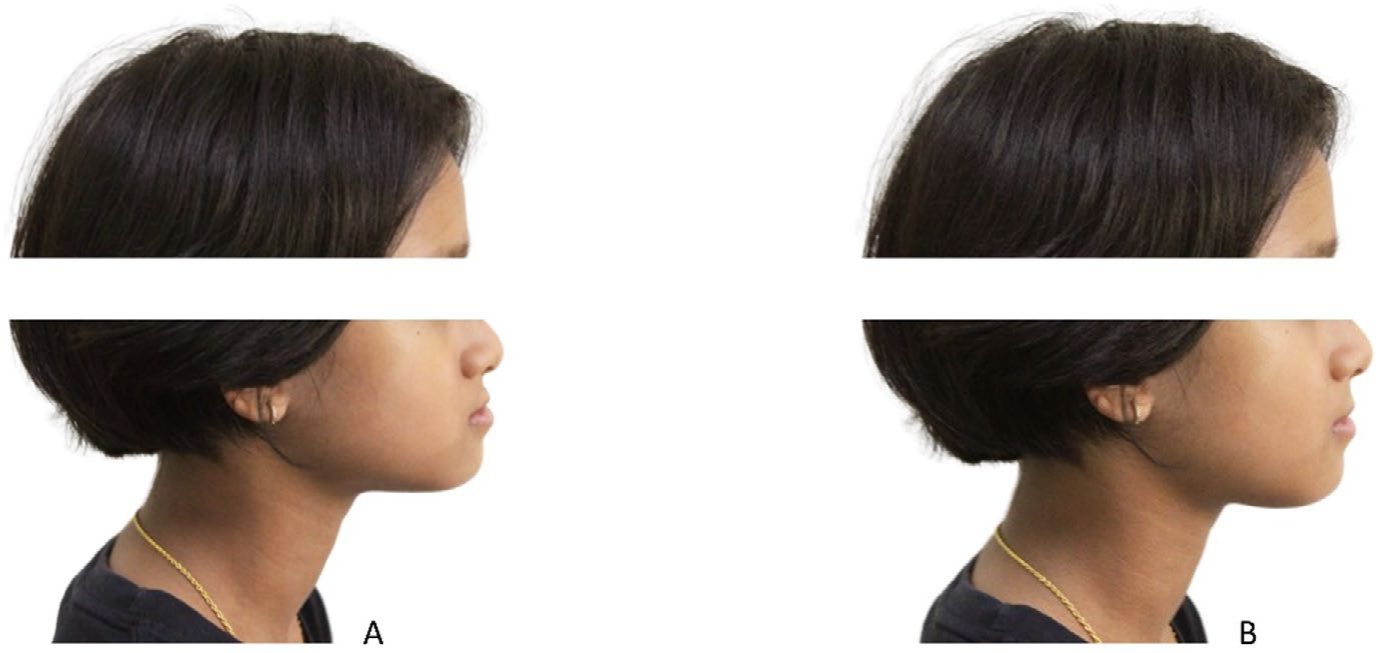
The Visual Treatment Objective. (A) Profile at maximum intercuspation. (B) Profile with the mandible at the retruded position.

The main treatment objectives included elimination of the premature contact and forward displacement of mandible, establishment of a positive overjet, and improvement of facial profile. The Visual Treatment Objective (VTO) was positive (Figure 6). The facial profile of the patient improved with the mandible in the most retruded position.

## 3 Methods

**FIGURE 7:**
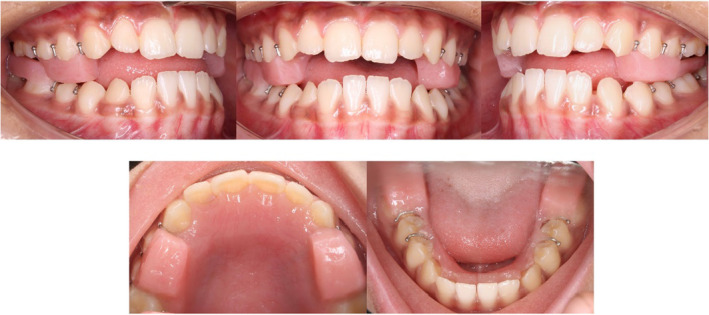
The Reverse Twin Block appliance inserted.

An RTB appliance was planned for the patient as she presented with a mild skeletal Class III pattern, had no crowding in either arch, and was in the prepubertal stage. Additionally, the patient expressed apprehension about fixed orthodontic treatment. For the fabrication of the appliance, bite registration was done with the mandible in the most retruded position with an inter‐incisal clearance of 2 mm and a clearance of 6 mm in the posterior segment. The biteblocks were inclined at 70° to the occlusal plane. The upper block covered the maxillary premolars, whereas the lower block covered the mandibular molars, thus enabling posterior positioning of the mandible (Figure 7). The appliance was fabricated from heat‐cure polymethyl methacrylate (Trevalon, Dentsply).

**FIGURE 8:**
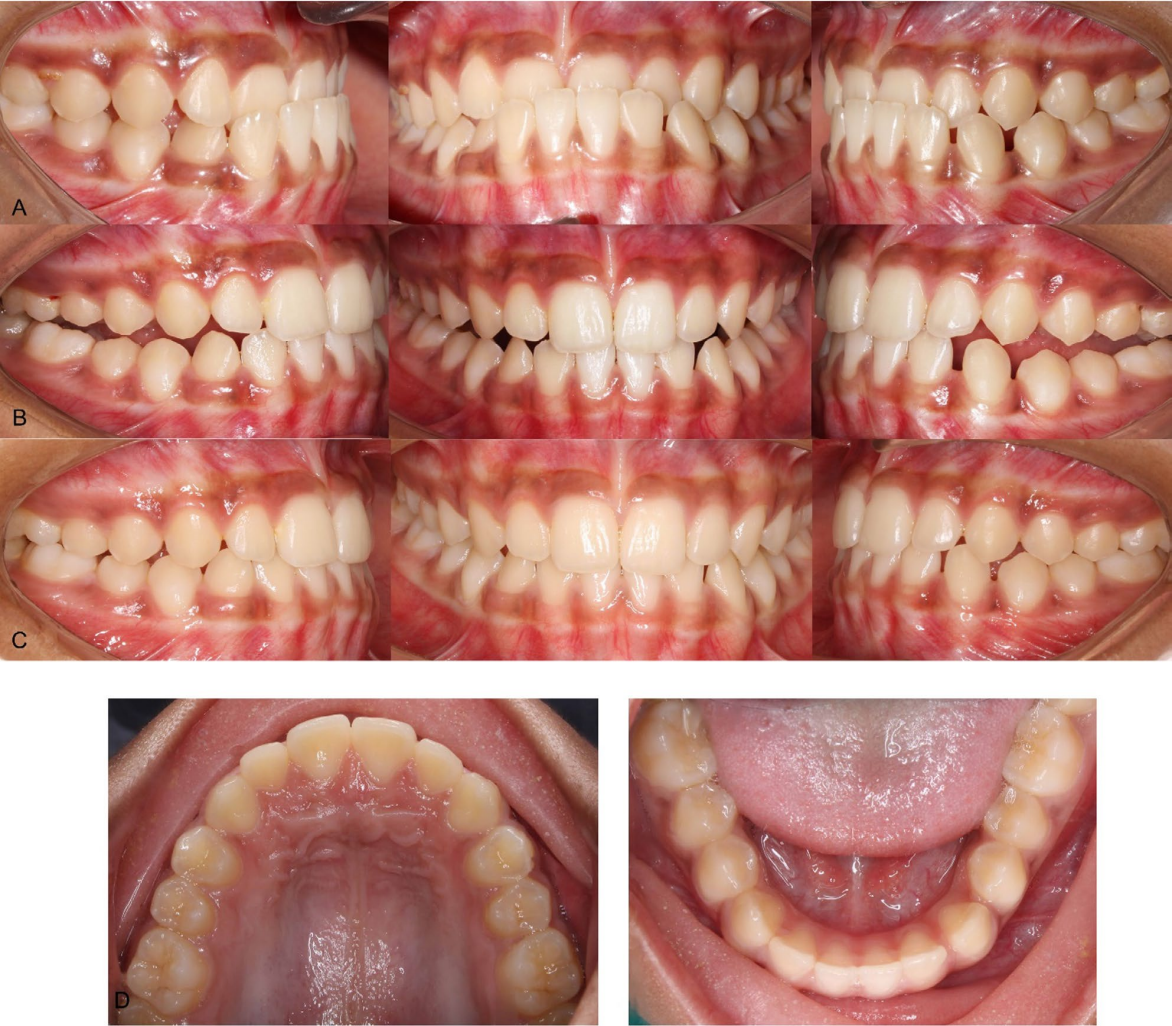
(A) Pre‐treatment occlusion at maximum intercuspation. (B) Occlusion after achieving positive overjet. (C) Occlusion at 1‐year follow‐up. (D) Maxillary and mandibular arches at 1‐year follow‐up.

The patient was instructed to wear the appliance full‐time except during meals and contact sports. Follow‐up visits were scheduled at 4‐week intervals to monitor treatment progress, compliance, and appliance retention. After 6 months of full‐time wear, positive overjet was achieved (Figure 8B). The patient was then instructed to wear the appliance only at night to allow for the settling of the posterior occlusion during the day.

## 4 Results

**FIGURE 9:**
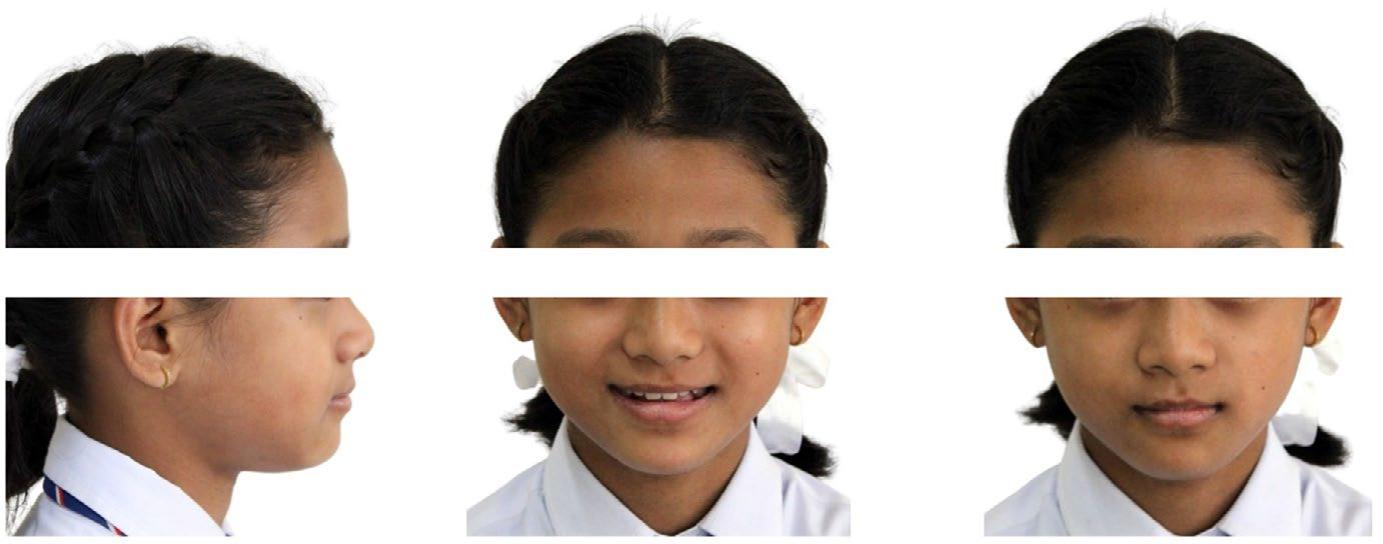
Post‐treatment extraoral photographs.

**FIGURE 10:**
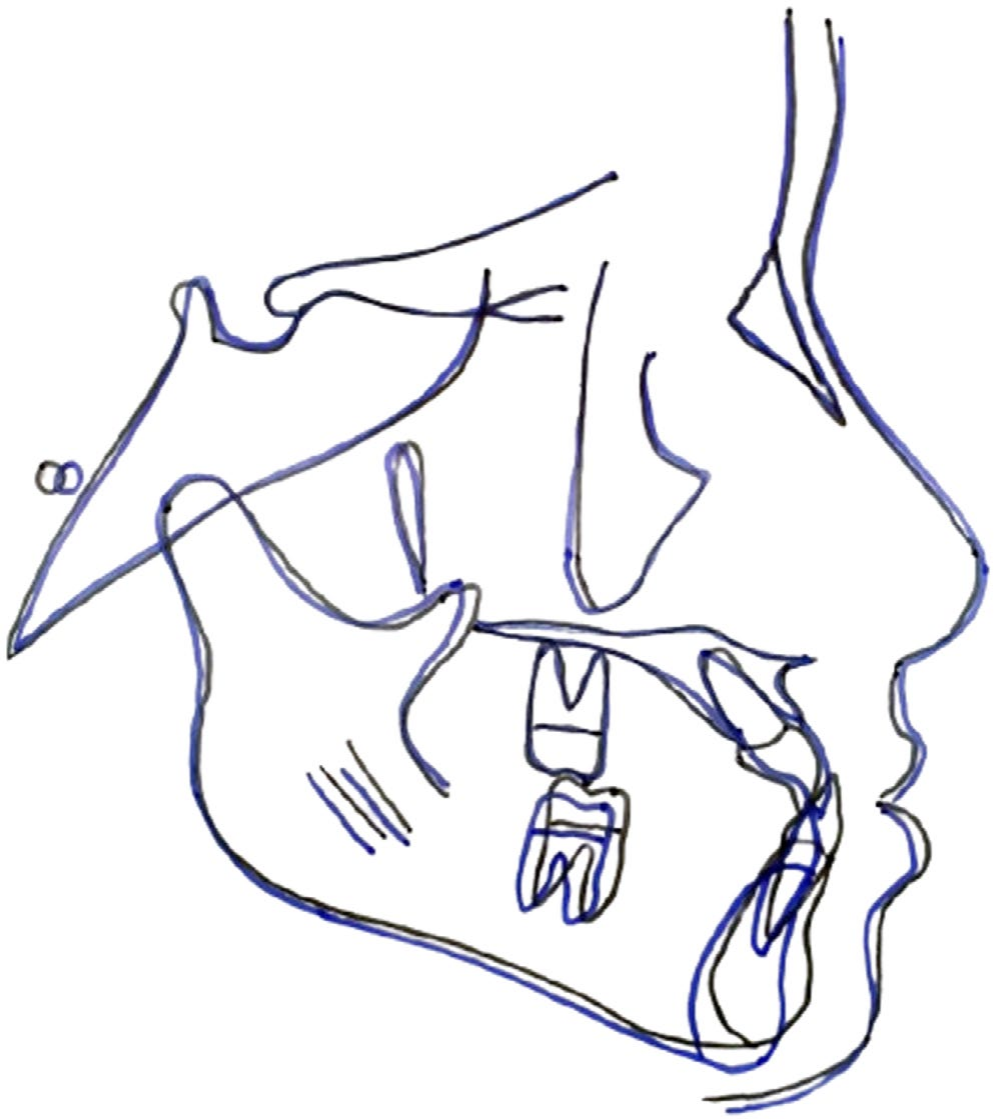
Superimposition of pre‐treatment (black) and post‐treatment (blue) cephalograms.

**FIGURE 11:**
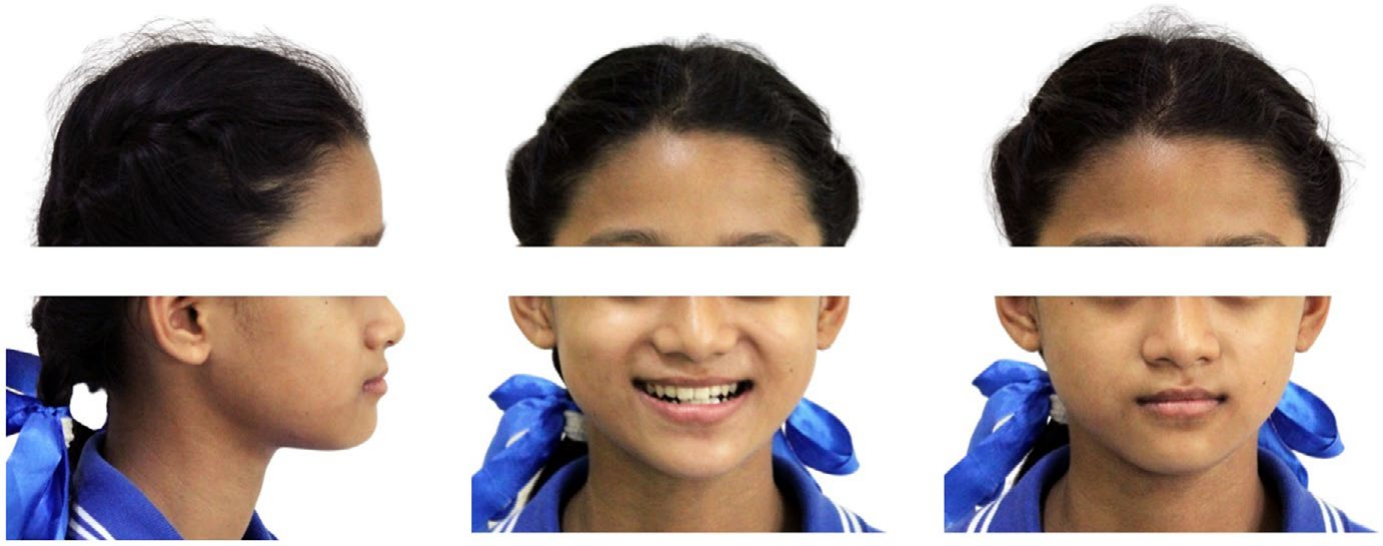
Extraoral photographs at 1‐year follow‐up.

After 8 months of treatment, the patient showed significant improvement in facial esthetics along with the establishment of positive overjet and normal overbite. Post‐treatment, the patient had a straight facial profile, Class I incisor relationship, Class I canine relationship, Class I molar relationship on the left, and Super‐class I molar relationship on the right side. A space of 1 mm was present both mesial and distal to mandibular left canine (Figures 8 and 9). Post‐treatment cephalogram revealed improvement in the sagittal relationship of jaws (Table 1) as well as improvement in the vertical relationship brought about by clockwise rotation of the mandible. There was proclination of the maxillary incisors and retroclination of the mandibular incisors (Figure 10). At 1‐year follow‐up, the positive overjet was maintained along with a straight facial profile (Figures 8C and 11).

## 5 Discussion

A Class III growth pattern is usually established early and typically deteriorates with age [13]. Early correction of a developing Class III malocclusion conveys both cosmetic and dental health benefits. Untreated anterior displacements have been associated with rapid periodontal destruction, accelerated occlusal wear, and temporomandibular joint disorder and have the potential to progress into a true skeletal class III malocclusion [14].

The RTB appliance utilizes inclined planes to harness occlusal forces, creating a reciprocal force system. Skeletally, the appliance promotes anterior displacement of the maxilla through stimulation of the circum‐maxillary sutures, while restricting mandibular growth and inducing a clockwise rotation of the mandible. Dentally, the appliance causes proclination of upper incisors and retroclination of lower incisors, along with mesial movement of upper molars and distal movement of lower molars [6, 7, 15]. These changes lead to the establishment of a positive overjet and overbite. Further, Clark [6] also reported that when the bite is maximally retruded, the distalizing force exerted on the mandibular condyles is not harmful, as the bite is hinged open with the condyles positioned down and forward in the fossae.

The superimposition of pre‐treatment and post‐treatment cephalogram showed similar favorable changes. The treatment effects were primarily dentoalveolar. The maxillary incisors were proclined by 3°, while the mandibular incisors were retroclined by 3°. These findings are consistent with those of Kidner et al. [15] and Seehra et al. [7] Likewise, Singh et al. [16] and Shastri et al. [17] also reported similar changes; however, they used fixed orthodontic treatment in conjunction with the RTB appliance. The RTB appliance also controlled and stabilized the growth of the mandible by eliminating forward slide, thereby resulting in clockwise rotation of the mandible. Similar effects on the mandible have been reported by other studies as well [7, 9, 15, 16]. As the effects of the RTB appliance are primarily dentoalveolar, it can be even used in patients who have passed their peak pubertal growth. Singh et al. [16] successfully used the RTB appliance along with fixed appliance to treat class III malocclusion in post‐pubertal patients.

Seehra et al. [7] recommended the factors governing the use of the RTB, which included the patient's age and the skeletal and occlusal relationships. The ideal patient should be between 8 and 10 years old and in the mixed dentition. Skeletal indicators include a mild Class III skeletal pattern due to mild mandibular prognathism with a normal or mildly retrognathic maxilla, with an average or below‐average maxillary‐mandibular plane angle and lower facial height. Dental indicators include reverse overjet with multiple teeth in cross‐bite, excessive overbite, minimal incisor compensation, anterior mandibular displacement on closure, and edge‐to‐edge incisor relationships in RCP. In such cases, the RTB appliance can be a good alternative to fixed orthodontic treatment. The RTB, being removable, offers greater convenience for eating and oral hygiene, potentially shorter treatment times, and lesser financial burden in comparison to fixed orthodontic appliance. However, it is not suited for complex cases and its effectiveness relies heavily on patient compliance.

A retrospective study by Seehra et al. [2] comparing the effects of RTB appliance and protraction headgear combined with rapid maxillary expansion for early correction of Class III malocclusion reported that both were effective treatment modalities but, compared to the protraction headgear, the RTB resulted chiefly in dentoalveolar changes, with greater proclination of the upper incisors and retroclination of the lower.

In our case, the patient was treated before her pubertal growth spurt, so unfavorable future growth could lead to reestablishment of a Class III malocclusion. Despite the chances, timely intervention helps eliminate anterior mandibular displacements and redirect jaw growth favorably. This subsequently reduces the need and extent of surgical procedures in the future [5]. There are many published literatures [7, 9, 15, 16], which demonstrate favorable short‐term changes with this appliance. However, long‐term stability and treatment effects remain largely unknown, highlighting the need of further research is needed in this aspect of the appliance.

## 6 Conclusion

The RTB is a simple and well‐tolerated appliance that can effectively correct developing Class III malocclusion in carefully selected cases. The primary effects are dentoalveolar with proclination of maxillary incisors and retroclination of mandibular incisors. In selected cases, this appliance can be a good alternative to fixed orthodontic treatment if timely intervention is done.

## Author Contributions

**Sushant Pandey:** conceptualization, data curation, methodology, writing – original draft. **Rajesh Gyawali:** methodology, supervision, writing – review and editing. **Prabhat Ranjan Pokharel:** methodology, supervision, writing – review and editing. **Avinash Chaudhary:** methodology, supervision, writing – review and editing. **Samikshya Sangroula:** methodology, supervision, writing – original draft.

## Consent

Written informed consent was obtained from the patient's mother to publish this report in accordance with the journal's policy.

## Conflicts of Interest

The authors declare no conflicts of interest.

## Data Availability

The data supporting the findings of this report are available from the corresponding author upon reasonable request.
